# Point-of-Care Screening for a Current Hepatitis C Virus Infection: Influence on Uptake of a Concomitant Offer of HIV Screening

**DOI:** 10.1038/s41598-018-33172-w

**Published:** 2018-10-17

**Authors:** Anna Maria Geretti, Harrison Austin, Giovanni Villa, Dan Hungerford, Colette Smith, Paula Davies, Jillian Williams, Apostolos Beloukas, Wojciech Sawicki, Mark Hopkins

**Affiliations:** 1 Institute of Infection and Global Health, University of Liverpool, Liverpool, United Kingdom; 2 Department of Infection and Population Health, University College London, London, United Kingdom; 3 Royal Liverpool University Hospital, Liverpool, United Kingdom; 4 Barts Health NHS Trust, London, United Kingdom

## Abstract

Eliminating hepatitis C as a public health threat requires an improved understanding of how to increase testing uptake. We piloted point-of-care testing (POCT) for a current HCV infection in an inner-city Emergency Department (ED) and assessed the influence on uptake of offering concomitant screening for HIV. Over four months, all adults attending ED with minor injuries were first invited to complete an anonymous questionnaire then invited to test in alternating cycles offering HCV POCT or HCV+HIV POCT. Viral RNA was detected in finger-prick blood by GeneXpert. 814/859 (94.8%) questionnaires were returned and 324/814 (39.8%) tests were accepted, comprising 211 HCV tests and 113 HCV+HIV tests. Offering concomitant HIV screening reduced uptake after adjusting for age and previous HCV testing (odds ratio 0.51; 95% confidence interval [CI] 0.38–0.68; p < 0.001). HCV prevalence was 1/324 (0.31%; 95% CI 0.05–1.73); no participant tested positive for HIV. 167/297 (56.2%) POCT participants lived in the most deprived neighbourhoods in England. HCV RNA testing using finger-prick blood was technically feasible. Uptake was moderate and the offer of concomitant HIV screening showed a detrimental impact on acceptability in this low prevalence population. The findings should be confirmed in a variety of other community settings.

## Introduction

An estimated 1% of the world population is chronically infected with HCV and at risk of developing cirrhosis, liver failure, and hepatocellular carcinoma^1^. With growing availability of curative antiviral therapy, the World Health Organisation (WHO) has set the global target of eliminating HCV infection as a major public health threat by 2030^1^. In the United Kingdom (UK), around 214,000 people are living with chronic HCV infection, and populations who have poor healthcare outcomes, low healthcare system engagement (through access or behaviours), and are socioeconomically deprived, are disproportionately affected^2^. At best, about half of infected people are aware of their status, and Public Health England has called for increase testing efforts in order to meet the WHO target of 90% diagnosed by 2030^2^. In healthcare settings, one successful strategy has been to implement opt-out screening in all patients undergoing venous blood collection as part of routine Emergency Department (ED) care^3^. Point-of-care testing (POCT) using capillary blood collected by finger-prick circumvents the requirement for venepuncture, offering a preferred testing approach for people who do not require additional laboratory investigations: POCT by finger-prick has become well-established for diagnosing HIV infection both within and outside hospital settings^4,5^. In the case of HCV, point-of-care tests have typically relied on antibody detection with limited sensitivity, and required confirmation of carrier status by laboratory-based HCV RNA testing^6^.

Diagnostic algorithms for hepatitis C recommend screening for HCV antibodies followed by confirmation of active infection with a molecular assay targeting HCV RNA^7^. As maturation of antibody responses occurs slowly after transmission, direct screening for HCV RNA substantially reduces the diagnostic window^8^. Until recently, molecular testing for the detection of viral nucleic acid was confined to specialised laboratory facilities, requiring highly trained personnel and a rigorous application of measures to prevent contamination during the separate steps of sample preparation and nucleic acid extraction, amplification and detection. Newer systems now combine the separate steps into a single, self-contained process that is easy to perform by non-specialist personnel and has a low risk of cross-contamination between specimens, thus offering a major diagnostic advance^9–12^.

Although the epidemic patterns are only partially overlapping in the UK, HCV and HIV share transmission routes and risk factors, and there is advocacy and precedent for combined screening programmes^3,13^. For effective roll-out, in addition to addressing the practical difficulties associated with engaging hard to reach populations, social and cultural barriers must be taken into account. Stigma - an attribute that links a person to an undesirable stereotype - is well described in relation to HIV infection^14,15^, and has been proposed to influence testing decision^15^. This study examined the feasibility of offering HCV RNA screening at point of care using small-volume capillary blood collected by finger-prick among attendees of an inner city ED, targeting adults presenting with minor injuries and not undergoing venepuncture for their routine care. We used the GeneXpert cartridge-based real-time molecular system, after establishing performance and simplicity of use in a validation study. The platform allows samples to be run individually without a requirement for batch testing and also allows multiple different tests to be run at the same time. The key aim of the study was to evaluate the impact on POCT acceptance of offering HCV screening alone relative to the offer of screening for both HCV and HIV, and thus gain insights into how the offer of POCT should be articulated to optimise uptake.

## Methods

### Finger-prick procedure

Capillary blood was collected by trained personnel following established procedure^16^, using Accu-Check Safety Safe-T-Pro Plus lancets (Roche, Burgess Hill, UK) and Microvette 100 K3 EDTA collection tubes (Sarstedt, Leicester, UK).

### Small-volume and large-volume protocols

HCV was detected by Xpert HCV Viral Load (Cepheid, Sunnyvale, CA), which provides a quantitative HCV RNA result. In the large-volume protocol, 1.1 ml of sample was directly added to one cartridge as recommended by the manufacturer. In the small-volume protocol, 1 ml of Xpert diluent was added to one cartridge, followed by 100 µl of sample (equating to a 1:11 dilution). The manufacturer reports that with the large-volume protocol the assay has a lower limit of quantification (LLQ) and a lower limit of detection (LLD) of 10 and 4 IU/ml, respectively. With the small-volume protocol applied in this study, based on the 1:11 dilution, the expected LLQ and LLD were 110 IU/ml and 44 IU/ml, respectively. HIV-1 was detected by the Cepheid Xpert HIV-1 Qual assay, which detects total HIV-1 nucleic acid (DNA and RNA) and provides a qualitative result (detectable or undetectable) with a LLD of 350 copies/ml^17^. The manufacturer’s established small-volume protocol for HIV-1 recommends adding 750 µl of diluent to a cartridge followed by 100 µl of sample. Each cartridge was loaded individually on the Cepheid GeneXpert XVI platform, with a total run time of 105 minutes for HCV and 90 minutes for HIV. Results were analysed with MedCalc v17.2 software (MedCalc, Belgium).

### Validation

The validation phase established assay performance with small-volume samples in three experiments. Firstly, HCV RNA detection in capillary blood was compared to detection in venous blood using paired samples obtained from 50 patients whose HCV status was already known, and including 39 HCV RNA positive and 11 HCV RNA negative subjects (Supplementary Table 1). This part of the study was approved by the NHS Research Authority (HRA; ref 15/NW/0715) and carried out in accordance with Good Clinical Practice guidelines; all participants provided written informed consent. Capillary blood was collected by finger-prick as described. Venous blood was collected in Monovette EDTA KE/2.7 mL tubes (Sarstedt). Paired capillary and venous blood samples were processed by laboratory personnel blinded to the patient’s status. Plasma was separated by centrifugation of venous blood at 4,500 g for 5 minutes and processed according to the large-volume protocol; capillary blood was processed in parallel according to the small-volume protocol. When comparing capillary blood with plasma, there was 100% agreement in qualitative HCV RNA detection across plasma HCV RNA levels ranging from 158 to 5,011,872 IU/ml. There was good agreement for quantitative HCV RNA results, with only two samples (genotype 3a) falling outside the 95% levels of agreement in the Bland-Altman analysis: one was under-quantified in capillary blood and one in plasma (Supplementary Fig. 1). In a second experiment, a reference panel with known HCV RNA load representing HCV genotypes 1a, 1b, 2b, 3a, 4a, 4d, 5a, and 6a was tested according to the small-volume protocol. Serial dilutions were prepared in basematrix (SeraCare, Milford, USA) ranging down to 2 log_10_ IU/ml, and from each dilution point 100 µl were tested in duplicate (Supplementary Fig. 2). HCV RNA was qualitatively detected in all 82 samples. Among 24 samples representing the lowest dilution point of 2 log_10_ IU/ml, 12 (50%) yielded quantified HCV RNA levels (median 189 IU/ml; range 145–355 IU/ml); the other 12 samples yielded qualitative HCV RNA detection. Good linearity (R^2^ = 0.90) was observed for measured vs. expected HCV RNA levels. In the third experiments, to determine the stability of HCV RNA detection in small-volume samples, whole venous blood collected from a HCV antibody and RNA negative individual was spiked with the 4^th^ International Standard for HCV RNA (HCV genotype 1a; NIBSC, Potters Bar, UK) and 100 µl aliquots were prepared containing 100 IU/ml of HCV RNA. The aliquots were stored at room temperature and away from light for 1, 3, 7, 10, 15, and 50 days, and then tested in duplicate according to the small-volume protocol. Based on the LLQ of 110 IU/ml, only qualitative detection HCV RNA was expected; HCV RNA was qualitatively detected in all 12 samples, thus demonstrating stability.

### Implementation

#### Setting

Implementation took place at the ED of the Royal Liverpool University Hospital (RLUH), the largest hospital in Merseyside, England, offering a 24-hour, seven days a week walk-in service. Merseyside is a large metropolitan area containing the city of Liverpool and has an estimated resident population of 1.4 million. In 2016/2017 there were 109,927 attendances at the RLUH ED, mostly (90%) by Merseyside residents and predominantly (79%) from the city of Liverpool^18^. Socio-economic deprivation is variable in Merseyside, but the area contains some of the most deprived neighbourhoods in England, with over 60% of its population living in a more deprived area than the average for England^19,20^.

#### Ethics and Approvals

The study received ethical approval from the NHS Research Authority (HRA REC. 16/LO/2007). The study protocol was approved by the University of Liverpool NHS HRA. The study was carried out in accordance with all Good Clinical Practice guidelines.

#### Study plan

**Figure 1:**
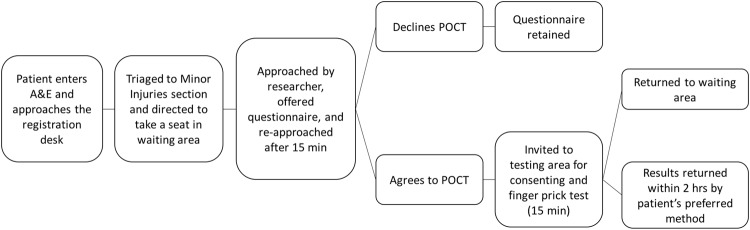
Flow of participant recruitment in ED.

The study was conducted in three cycles of 4, 2 and 2 weeks’ duration respectively, each separated by a pause of one week (Fig. 1). Each cycle comprised one phase offering HCV POCT alone, followed by a phase offering HCV and HIV POCT combined. Testing was offered in blocks of 7 hours that on each day started at different times of the day to ensure the whole 24 hours were covered. Within each cycle, the timing structure of the two phases (HCV and HCV+HIV) was kept identical.

#### Questionnaires

Upon entering via the dedicated ED entrance of the hospital, walk-in patients reported to reception for registration and triage. For the purpose of the study, at registration, using information material tailored to the testing phase (HCV alone or HCV+HIV) (Supplementary Note Participant Information Sheet), all adults (≥18 years) triaged as attending with minor injuries were offered an information sheet detailing the benefits of early diagnosis and the availability and efficacy of treatment. Attendees were then invited to complete an anonymous questionnaire (Supplementary Note Questionnaire), which collected age, gender, ethnicity, and awareness of any previous HCV test. The questionnaire also offered the option to accept or decline POCT, and if POCT was declined, asked for the reasons of refusal. Participants were informed that the test would not impair their routine care.

#### Testing

If testing was accepted, trained study personnel took participants to a testing area in ED. At this stage, written informed consent was taken prior to testing and participants were also asked to indicate their residential postcode and preferred method for receiving results, choosing among waiting for face-to face delivery within two hours, receiving a phone call, or receiving a standardised text message from a dedicated study phone stating either “No follow-up required” or “Contact study nurse for follow-up”. Patients were advised that should the POCT be reactive, access to immediate advice by a specialist nurse would be available and a follow-up appointment would be arranged for confirmation of test results and specialist evaluation to take place within two days for HIV and two weeks for HCV. Capillary blood was obtained by finger-prick as described, and the same personnel processed the sample on site according to the small-volume protocol using Xpert HCV Viral Load and Xpert HIV-1 Qual.

### Socio-economic and health deprivation profile of POCT participants based on area of residence

Postcodes provided by POCT participants were extracted from the study record forms without patient identifiers and matched to English Lower layer Super Output Areas (LSOAs) using GeoConvert^21^. LSOAs are statistical geographical areas containing approximately 1,500 people^20^. LSOAs were matched to the 2015 English indices of deprivation to assign residential postcodes to national deprivation deciles^19^. The most commonly used measure of the deprivation in England is the Index of Multiple Deprivation (IMD), a weighted score constructed from seven domains including: Income, Employment, Education skills and Training, Health and Disability, Crime, Barriers to housing and services, Living environment, and Other service^19^.

### ED staff interviews

At the end of the study, one-to-one interviews were conducted with four senior ED Nurse Practitioners, the primary care providers in the minor injuries section, using a standard set of questions (Appendix III).

### Statistical analysis

Pearson’s chi square test was used to compare stated reasons for declining POCT between total HCV and total HCV+HIV phases. Factors associated with POCT uptake were analysed by univariable and multivariable logistic regression. Variables included in the univariable analysis comprised self-reported age (divided into age groups 18–34, 35–54, 55–64, and 65+), gender, ethnicity and HCV testing history; time of ED attendance (morning 6 am-noon, afternoon noon-6 pm, and evening/night 6 pm-6 am); and POCT phase (HCV alone or HCV+HIV). Variables showing an association with p-value < 0.2 in the univariable analysis were carried forward in the multivariable model. All analyses were conducted in STATA version 14.1 (College Station, TX, USA).

### Patient involvement

Patients’ experience with the finger-prick procedure was evaluated during the validation phase of the study, which recruited patients with known HCV, hepatitis B virus (HBV) and HIV status attending the Infectious Diseases Service of the RLUH. Results of the implementation study are to be discussed with community representatives within the British HIV Association and further disseminated in collaboration with the Hepatitis C Trust. Follow-up focus group studies are planned to discuss attitudes to HIV testing in the local population.

### Transparency Declaration

Anna Maria Geretti affirms that the manuscript is an honest, accurate, and transparent account of the study being reported; that no important aspects of the study have been omitted; and that any discrepancies from the study as planned have been explained.

### Sponsorship

This study was sponsored jointly by the University of Liverpool and the Royal Liverpool University Hospital.

## Results

### Questionnaires

Between March 2017 and June 2017, 814/859 subjects (94.8%) returned a completed questionnaire (Table 1).

**Table 1:** Characteristics of questionnaire respondents according to POCT uptake.

		Responded	POCT	
			Accepted	Declined
Total population, n (%)		814 (100)	324 (100)	490 (100)
Age	18–34, n (%)	399 (49.0)	158 (48.8)	241 (49.2)
	35–54, n (%)	248 (30.5)	93 (28.7)	155 (31.6)
	55–64, n (%)	89 (10.9)	47 (14.5)	42 (8.6)
	65+, n (%)	72 (8.9)	26 (8.0)	46 (9.4)
	Not given, n (%)	6 (0.7)	0 (0.0)	6 (1.2)
Gender	Female, n (%)	401 (49.3)	160 (49.4)	241 (49.2)
	Male, n (%)	411 (50.5)	164 (50.6)	247 (50.4)
	Not given, n (%)	2 (0.2)	0(0)	2 (0.4)
Ethnicity	White, n (%)	725 (89.1)	292 (90.1)	433 (88.4)
	Other, n (%)	87 (10.7)	32 (9.9)	55 (11.2)
	Not given, n (%)	2 (0.2)	0 (0.0)	2 (0.4)
Previous HCV test	No, n (%)	551 (67.7)	208 (64.2)	343 (70.0)
	Yes, n (%)	120 (14.7)	35 (10.8)	85 (17.3)
	Unsure, n (%)	143 (17.6)	81 (25.0)	62 (12.7)
Time of attendance	Afternoon, n (%)	401 (49.2)	155 (47.8)	246 (50.2)
	Morning, n (%)	261 (32.1)	106 (32.7)	155 (31.6)
	Evening/night, n (%)	152 (18.7)	63 (19.4)	89 (18.2)

POCT = Point-of-care testing.

Respondents attended predominantly in the afternoon. Most were aged below 55 years and of white ethnicity, with a similar proportion of men and women. Among a subset (120/814, 14.7%) reporting a previous HCV test, 7/120 (5.8%) and 110/120 (84.2%) indicated a positive and a negative test result, respectively; 12/120 (10.0%) were not sure of the result. Among the 814 respondents, 451 (55.4%) were offered HCV POCT and 363 (44.6%) were offered combined HIV+HCV POCT. Overall, 211/451 (46.8%) and 113/363 (31.2%) consented to HCV POCT and HCV+HIV POCT, respectively. When analysing the total population, reasons given for declining POCT showed no significant difference between HCV phases vs. HCV+HIV phases. The most common reasons given for declining POCT was inconvenience (Table 2).

**Table 2:** Questionnaire and test uptake by cycle and phase, and reasons given for declining POCT.

	Total population, n (%)	All Cycles, n (%)		Cycle 1, n (%)		Cycle 2, n (%)		Cycle 3, n (%)	
		HCV phases	HCV+HIV phase	HCV phase	HCV+HIV phase	HCV phase	HCV+HIV phase	HCV phase	HCV+HIV phase
Questionnaires offered, n (%)	859 (100)	471 (100)	388 (100)	184 (100)	121 (100)	147 (100)	112 (100)	140 (100)	155 (100)
Questionnaire completed, n (%)	814 (94.8)	451 (95.7)	363 (93.5)	177 (96.1)	100 (82.6)	135 (91.8)	108 (96.4)	139 (99.3)	155 (100)
Accepted POCT^a^, n (%)	324 (39.8)	211 (46.8)	113 (31.2)	90 (51.9)	34 (34)	70 (51.9)	41 (38.0)	51 (36.7)	38 (24.5)
Declined POCT, n (%)	490 (60.2)	240 (53.2)	250 (68.8)	87 (49.1)	66 (66)	65 (48.1)	67 (62.0)	88 (63.3)	117 (75.5)
Reasons for declining POCT^b^, n (%)	567 (100)	264 (100)	303 (100)	97 (100)	83 (100)	74 (100)	81 (100)	93 (100)	139 (100)
I am worried/I do not wish to know, n (%)	22 (3.9)	9 (3.4)^c^	13 (4.3)	4 (4.1)	7 (8.4)	1 (1.4)	2 (2.5)	4 (4.3)	4 (2.9)
I don’t want others to know I am being tested, n (%)	5 (0.9)	0 (0.0)	5 (1.7)	0 (0.0)	2 (2.4)	0 (0.0)	1 (1.2)	0 (0.0)	2 (1.4)
I don’t like the idea of a HIV test, n (%)	4 (0.7)	0 (0.0)	4 (1.3)	0 (0.0)	2 (2.4)	0 (0.0)	1 (1.2)	0 (0.0)	1 (0.7)
I don’t like blood tests, n (%)	89 (15.7)	46 (17.4)^d^	43 (14.2)	17 (17.5)	12 (14.5)	10 (13.5)	9 (11.1)	19 (20.4)	22 (15.8)
Time is not convenient, n (%)	175 (30.9)	88 (33.3)^e^	87 (28.7)	29 (29.9)	22 (26.5)	26 (35.1)	29 (35.8)	33 (35.5)	36 (25.9)
I was previously tested, n (%)	94 (16.6)	43 (16.3)^f^	51 (16.8)	19 (19.6)	15 (18.1)	14 (18.9)	16 (19.8)	10 (10.8)	20 (14.4)
I believe I am not at risk, n (%)	139 (25.4)	63 (25.8)^g^	76 (25.1)	26 (26.8)	17 (20.5)	16 (21.6)	16 (19.8)	26 (28.0)	43 (30.9)
Not stated, n (%)	35 (6.0)	10 (3.8)^h^	24 (7.9)	2 (2.1)	6 (7.2)	7 (9.5)	7 (8.6)	1 (1.1)	11 (7.9)

^a^Among questionnaire respondents; ^b^Among participants who declined testing, the number of reasons indicated surpassed the number of individuals declining, as participants were able to indicate more than one reason; for the total population, reasons for declining were compared between HCV phases vs. HCV+HIV phases with the following results: ^c^p = 0.59; ^d^p = 0.29; ^e^p = 0.23; ^f^p = 0.86; ^g^p = 0.74; ^h^p = 0.38 (Pearson’s chi square test). POCT = Point-of-care testing.

Factors associated with POCT uptake were analysed by univariable and multivariable regression analysis (Table 3).

**Table 3:** Factors associated with POCT uptake.

		Univariate			Multivariable^a^		
		OR	95% CI	p-value	OR	95% CI	p-value
Age group	18–34	1	—	0.06	1	—	0.03
	35–54	0.92	0.66–1.26		1.1	0.75–1.50	
	55–64	1.71	1.07–2.70		2.1	1.22–3.19	
	65+	0.90	0.51–1.45		0.9	0.54–1.60	
Gender	Female vs. Male	0.99	0.75–1.32	0.99			
Ethnicity	White vs. Non-White	1.15	0.73–1.83	0.53			
Time of attendance	Afternoon	1	—	0.80			
	Morning	1.08	0.78–1.49				
	Evening/Night	1.12	0.76–1.64				
Previous HCV test	No	1	—	<0.001	1	—	<0.001
	Yes	0.67	0.44–1.04		0.74	0.47–1.15	
	Unsure	2.15	1.48–3.12		2.36	1.60–3.48	
Phase of POCT	HCV vs. HCV+HIV	0.51	0.38–0.68	<0.001	0.51	0.37–0.69	<0.001

^a^Variables associated with POCT uptake in the univariate analysis with p-value < 0.2 were carried forward in the multivariable model. POCT = Pont-of-care testing.

In the univariable analysis, age group 55–64 years and lack of awareness of a previous HCV test were associated with increased uptake, whereas a concomitant offer of HIV POCT relative to the offer of HCV POCT alone significantly reduced uptake. Following adjustment for age and previous HCV testing, the concomitant offer of HIV POCT remained a significant predictor of reduced HCV POCT uptake.

### Testing

For POCT participants, the preferred method for receiving results was text messaging (260/324, 80.3%), whereas face-to-face delivery (33/324, 10.1%) and receiving a phone call (31/324, 9.7%) were less utilised. Overall 1/324 (0.31%) HCV tests and 4/113 (3.5%) HIV tests gave invalid results. Participants whose test gave an invalid result were offered immediate retest; those who declined a retest were given the details of how to access testing elsewhere. In total, 1/324 POCT participants tested HCV RNA positive, giving a HCV prevalence of 0.31% (95% CI 0.05 to 1.73). Among those screening for HIV, 0/109 were positive, yielding a HIV prevalence of 0.0% (95% CI 0.00 to 3.40).

### Population socio-economic deprivation

Among the 324 participants who accepted POCT, 297 (91.7%) had postcodes which could be matched to those published by the UK DataService Census Support. The majority of POCT participants (231/297, 77.8%) were from Liverpool neighbourhoods, followed by a smaller portion (38/297, 12.8%) from other Merseyside areas, and the remainder (28/297, 9.4%) from outside of the region. The IMD for the study cohort indicated that 167/297 (56.2%) of POCT participants lived in the most deprived (deciles 1 and 2) LSOAs in England. Stratifying the IMD score by the seven domains (Supplementary Table 2) showed that 221/297 (74.4%) participants fell within the most deprived areas for Health and Disability, 169/297 (56.9%) for Employment, and 159/297 (53.5%) for Income.

### ED staff focus interviews

Key aspects of the feedback comprised that no negative comments had been received from patients about the POCT experience, and that staff had suffered no negative impact on daily practice. Staff members expressed an overall positive view of the initiative and reported increased awareness around the importance of HCV and HIV testing, while also emphasising that adoption in routine practice would require additional, dedicated staff. Importantly, they reported that the initiative had improved their understanding that HCV carrier status may occur in the absence of symptoms.

## Discussion

This study determined the feasibility of offering point-of-care screening for HCV RNA in an inner-city Emergency Department using small-volume capillary blood obtained by finger-prick and investigated whether a concomitant offer of HIV screening had an influence on uptake. The approach proved technically feasible, and did not appear to affect care provision or the patients’ experience. There was a high return of the anonymous questionnaires offered at registration and prior to receiving information about the study. However, less than half of the respondents agreed to testing, and uptake was significantly reduced by the offer of a concomitant HIV test relative to the offer of HCV screening alone. These data point to the possibility that stigma and fears about HIV infection influenced behavioural patterns in this low-prevalence population, which was mostly composed of people of white ethnicity aged below 55 years from Merseyside in England.

We used the Cepheid GeneXpert system, a random-access, single-step nucleic acid amplification and detection platform which offers the advantage of modularity and therefore portability of small units. The GeneXpert platform has been used for the point-of-care diagnosis of infections such as gonorrhoea^11^ and influenza^12^. Applicability for HCV RNA screening by finger-prick was recently investigated among 210 subjects recruited from a variety of community settings in Australia (three drug and alcohol clinics, a homelessness service, and a needle and syringe programme)^22^. The study reported excellent sensitivity and specificity relative to the Abbott RealTime HCV Viral Load assay. Our validation data provide further technical information in support of the small-volume protocol by demonstrating good assay performance across the major HCV genotypes, a high degree of concordance with results obtained in paired venous blood, stability of HCV RNA detection over time, and a low failure rate when the assay was run by non-specialist healthcare personnel. A total of 5/437 (1.1%) point-of-care tests gave an invalid result, and the risk of failure can be reduced by ensuring sufficient volume of blood and diluent are added to the cartridge. Our validation data, together with those available from the literature, provide the required confidence to allow implementation of GeneXpert for HCV RNA testing at point of care across a variety of settings. It should be noted that using small-volume samples reduces assay sensitivity relative to testing a larger sample volume (estimated LLQ 110 IU/ml and LLD 44 IU/ml), which makes the finger prick technique not suitable for demonstrating HCV clearance soon after completion of antiviral therapy. Recent progress in cartridge technology have further shortened testing times on GeneXpert ensuring that individuals can receive results within one hour of sampling^23^. It should be noted however that in our study patients typically faced ED waiting times of at least two hours but nonetheless elected to receive POCT results by a text message. This is consistent with studies indicating that text messaging is the preferred method of result delivery among those consenting to POCT for HIV^24^. How ability to offer immediate face to face counselling to patients testing positive at POCT influences retention into follow-up remains to be determined.

Our population had a 95% rate of questionnaire return; however, test acceptance was less than half. Only one in seven questionnaire respondents was aware of their HCV status, although, promisingly, lack of awareness was an independent predictor of POCT uptake. The reasons given for declining POCT were not significantly different across phases, and inconvenience was the most commonly stated reason. Indeed, patients presenting to ED with acute injuries may wish to prioritise their immediate need even if facing a considerable wait. Only a small minority of those declining POCT in the HCV+HIV phases (4/250, 1.6%) stated that they would have a HCV test but did not like the idea of a HIV test. Nonetheless, the offer of a HIV test alongside the HCV test halved the odds of POCT uptake when compared to HCV testing alone, independently of age and previous HCV testing. These findings suggest that despite continued efforts to eradicate HIV stigma^25^, negative perceptions about HIV may have influenced willingness to undergo HCV testing in our cohort. This potential effect has been previously contemplated in an interview-based study^15^, but not formally demonstrated to date. In fact, the findings are contrary to studies showing that individuals often attempt to hide their interest in having a HIV test by undergoing other less stigmatized tests at the same time^26^. With the WHO backing the use platforms such as GeneXpert for HIV and HCV screening^27^, and advocacy for integrated testing strategies to improve efficiency and decrease cost^15,28^, the design of screening programmes should keep this potential pitfall into consideration, especially where opt-in strategies are pursued or indeed preferred by the targeted population^15^. Further studies are needed to determine whether these findings are generalizable across different populations of the UK and beyond, and whether the language used to encourage POCT participation and educational initiatives may help address the barrier.

At 0.31%, the prevalence of HCV infection was overall consistent with the estimated prevalence of 0.4% for England^29^. It should be noted that the study screened for a current hepatitis C infection, whereas estimated prevalence rates are typically based on antibody detection, which does not differentiate between a current and a past infection. Prevalence can drop by half when people with a past infection are excluded^3^. Socio-economic and health deprivation has been associated with an increased risk of HCV infection^30^, and we recently reported that the prevalence of a current HCV infection was as high as 10.4% among people using homeless hostels in London^31^. In this study, a large proportion of POCT participants were living in the most deprived neighbourhoods in England. Nonetheless, HCV prevalence was lower than expected for North West England, and for ED attendees undergoing opt-out testing of routinely collected venous blood in London^3,32^. This may reflect the composition of the study population that agreed to testing, and the lack of pre-screening for risk factors, mainly a current or past injecting drug use^29^. Data indicate that the age group 25–54 is a key target population for HCV screening in England^3,29^ and that middle-aged men are disproportionally affected in the North West^28^. In this study, 46/324 (14.2%) of those who agreed to testing were men aged 35–54 years, and of these 1/46 (2.2%) tested HCV positive. HIV prevalence in this study was low and in line with prevalence rates of around 0.2% for the North-West of England^33^. The overall number of new HIV diagnoses has declined in the region in recent years, and rates are influenced by the number of new diagnoses among black Africans^33^, an ethnic group that was not well represented in our study. Thus, the low estimated prevalence of HIV infection was consistent with the available epidemiological data. Given that HIV diagnoses often occur late in the North-West^33^, continued efforts to roll-out HIV testing are warranted.

This study is the first to implement testing for a current HCV infection within an ED in the UK using molecular testing directly delivered at point of care. One strength was the willingness of patients to engage, leading to a high return of questionnaires. To help eliminate bias, the dialogue used when initially approaching patients was kept succinct and consistent throughout the study, and completion of the anonymous questionnaires took place prior to provision of information about the study and formal consenting. Patients were recruited on each day of the week and on each hour of the day, allowing for a wide representation of the population attending for walk-in care. Importantly, within each cycle, the structure of the phases offering HCV POCT or HCV+HIV POCT was kept identical to preserve comparability. There are limitations to this study. This was a single-centre study addressing a selected population, that whilst representative of the local population, was rather homogenous in terms of ethnic background. Future studies would benefit from a multi-centre approach to capture population variances, with the specific aim of confirming and characterising the possible negative effect on POCT uptake of offering a concomitant HIV test. The sample size was sufficient to demonstrate the impact of offering HIV testing alongside HCV testing on overall acceptance. Prevalence of HCV and HIV was low in this population and further studies are required to investigate whether a similar effect is seen among populations with high HCV or HIV prevalence. The validation data we provide indicate that expanding the technique to non-hospital locations, including primary care, prisons, and community settings, is entirely feasible. In addition, we used measures of deprivation to describe how the tested population ranked when compared to other areas in England and future studies should aim to dissect the possible relationships between deprivation and POCT uptake, although we expect this to be inherently linked to healthcare system engagement.

Hepatitis C is curable. To reduce the large proportion of undiagnosed carriers, screening for HCV RNA can now be achieved at point of care with high sensitivity using a few drops of blood collected by finger-prick. Technical improvements are on the way that will further shorten turn-around time, ensuring that results and appropriate care can be delivered promptly. However, careful implementation is required to improve uptake, while respecting any preference for opt-in versus opt-out strategies in the targeted population. There is an urgent need to verify that the concomitant offer of a HIV test reduces uptake of HCV screening, and to evaluate strategies that may overcome the apparent barrier.

## Electronic supplementary material

Supplementary Information
