# Supplementary material for: Point-of-Care Screening for a Current Hepatitis C Virus Infection: Influence on Uptake of a Concomitant Offer of HIV Screening
Source: Sci Rep. 2018 Oct 17;8:15297. doi: 10.1038/s41598-018-33172-w (PMC6193009; doi:10.1038/s41598-018-33172-w)
Supplement: Supplementary file 1 — Supplementary Information [file 41598_2018_33172_MOESM1_ESM.pdf]

Supplementary Information

Point-of-care screening for a current hepatitis C virus infection: influence on uptake of a concomitant offer of HIV screening

Anna Maria Geretti[a*], Harrison Austin[a^], Giovanni Villa[a^], Dan Hungerford[a], Colette Smith[b] Paula Davies[c], Jillian Williams[c], Apostolos Beloukas[a], Wojciech Sawicki[c], Mark Hopkins[c,d]

[a]Institute of Infection and Global Health, University of Liverpool, Liverpool, United Kingdom

[b]Department of Infection and Population Health, University College London, London, United Kingdom

[c]Royal Liverpool University Hospital, Liverpool, United Kingdom

[d]Barts Health NHS Trust, London, United Kingdom

^Authors contributed equally to the study

[*]Corresponding author

Professor Anna Maria Geretti

Dept. of Clinical Infection, Microbiology & Immunology

University of Liverpool

8 West Derby Street, L69 7BE, United Kingdom

+44 151 795 9665

geretti@liverpool.ac.uk

Supplementary Note Questionnaire

QUESTIONNAIRE 1

Please do not write your name or contact details here so that we can keep the information anonymous

1. What is your gender? Male ( ) Female ( )

2. What is your age? 18-24y ( ) 25-34y ( ) 35-44y ( ) 45-54y ( ) 55-64y ( ) 65y+ ( )

3. How would you describe your ethnic origin? *Tick one option only*

White British	☐	Black African	☐	Asian Other	☐
White other	☐	Black other	☐	Arabic	☐
Black British	☐	Asian British	☐	Mixed	☐
Black Caribbean	☐	Chinese	☐	Other ethnic background	☐

4. Have you ever had a hepatitis C test before? Yes ( ) No ( ) Not sure ( )

5. If you had a hepatitis C test in the past, what was the result?

Positive ( ) Negative ( ) Not sure ( )

6. Would you like to have a hepatitis C test today? Yes ( ) No ( ) Not sure ( )

7. If you do NOT want a hepatitis C test today, which is the reason? *Tick all that apply*

- I had a test in the last 5 years and it was negative ( )
- I already know I have hepatitis C ( )
- I don’t want to know if I have hepatitis C ( )
- The test is not convenient at this time ( )
- I don’t like blood tests ( )
- I am worried about what it means to get a reactive test ( )
- I don’t want other people to know I am having a hepatitis C test ( )
- I believe I am not at risk for hepatitis C ( )
- I believe I may be at risk for hepatitis C and I am worried ( )
- Other reason (please state below)

  ……

  ……

8. If you already know you have hepatitis C, which of these applies to you? *Tick all the apply*

- I have been seen by a liver specialist in the last 12 months ( )
- I have been seen by a liver specialist longer than 12 months ago ( )
- I have been treated for hepatitis C infection ( )
- I have been told I am now clear of the hepatitis C infection ( )
- I have been told I need treatment for hepatitis C but I do not feel ready ( )
- I have been meaning to contact my GP or the hospital to make an appointment with the liver clinic ( )

Thank you very much for your time

QUESTIONNIARE 2

Please do not write your name or contact details here so that we can keep the information anonymous

1. What is your gender? Male ( ) Female ( )

2. What is your age? 18-24y( ) 25-34y( ) 35-44y( ) 45-54y( ) 55-64y( ) 65y+ ( )

3. How would you describe your ethnic origin? *Tick one option only*

White British	☐	Black African	☐	Asian other	☐
White other	☐	Black other	☐	Arabic	☐
Black British	☐	Asian British	☐	Mixed	☐
Black Caribbean	☐	Chinese	☐	Other ethnic background	☐

4. Have you ever had a hepatitis C test before? Yes ( ) No ( ) Not sure ( )

5. If you had a hepatitis C test in the past, what was the result?

Positive ( ) Negative ( ) Not sure ( )

6. Would you like to have a hepatitis C test today? Yes ( ) No ( ) Not sure ( )

7. If you do NOT want a hepatitis C test today, which is the reason? *Tick all that apply*

- I had a test in the last 5 years and it was negative ( )
- I already know I have hepatitis C ( )
- I don't want to know if I have hepatitis C ( )
- The test is not convenient at this time ( )
- I don't like blood tests ( )
- I am worried about what it means to get a reactive test ( )
- I don't want other people to know I am having a hepatitis C test ( )
- I don't mind having a hepatitis C test but I don't like the idea of having a HIV test ( )
- I believe I am not at risk for hepatitis C ( )
- I believe I may be at risk for hepatitis C and I am worried ( )
- Other reason (please state below)

  ……

  ……

8. If you already know you have hepatitis C, which of these applies to you? *Tick all the apply*

- I have been seen by a liver specialist in the last 12 months ( )
- I have been seen by a liver specialist longer than 12 months ago ( )
- I have been treated for hepatitis C infection ( )
- I have been told I am now clear of the hepatitis C infection ( )
- I have been told I need treatment for hepatitis C but I do not feel ready ( )
- I have been meaning to contact my GP or the hospital to make an appointment with the liver clinic ( )

Thank you very much for your time

Supplementary Note: Participant Information Sheet

PARTICIPANT INFORMATION SHEET 1

The Royal Liverpool Hospital, in collaboration with the University of Liverpool, is trying out new ways to improve care. As part of this initiative you are being invited to take part in a research study concerning **hepatitis C**. We will offer a screening test for hepatitis C to all adults attending the A&E Minors department today. Please read the following information

What is hepatitis C?
Hepatitis C is an infection of the liver caused by the hepatitis C virus.

How do people catch hepatitis C?
The infection is typically transmitted through contact with infected blood.

How common is hepatitis C?
It is estimated that for every 100 people in Cheshire and Merseyside, four have hepatitis C.

What are the symptoms of hepatitis C?
Hepatitis C remains silent for many years before causing any symptoms and therefore people do not realise that they have the infection unless a test is done. After about 20 years, hepatitis C may cause scarring of the liver (cirrhosis), followed by liver failure requiring a transplant. Cancer of the liver may also develop. It is important to diagnose the infection before these complications occur so that they can be prevented.

Can hepatitis C be treated?
Yes, treatment is available that can cure the infection in over 90% of people. The Royal Liverpool Hospital is one of the major centres in England offering treatment for hepatitis C. Currently, treatment is prioritised according to clinical need.

For more information, you may find this website useful:
www.nhs.uk/conditions/Hepatitis-C

carefully.

What is this study about?
We are offering adult people attending the A&E department a blood test for hepatitis C. Our objectives are to encourage people to get tested and be diagnosed, and also to understand what people think about this way of offering a test. Your opinion and feedback are important to us, whether you decide to have the test or not.

How is the hepatitis C test done?
Our test uses a few drops of blood collected from a finger prick, instead of blood from a vein. The fingerprick takes 5 minutes. Most people find that it is not painful. The finger prick blood is then tested on a machine in A&E, and the results are ready after 2 hours.

Do I have to wait in A&E for the results?
You may wait if you wish. Alternatively, you can leave your phone number and as soon as the results are ready we will text or call you, depending on your preference.

Do I have to have the test?
We would like to encourage you to have the test. However, you are perfectly free to decline the test. Your decision about the test will not in any way affect the rest of your care today.

What next?

Whether you decide to have a test or not, please could you complete a **short questionnaire** *(see overleaf)*? Your opinion matters to us. It will help us improve the service we offer. The information given in the questionnaire will be completely anonymous and will not be traceable back to you.

What will happen to information stored about me?

If you decide to take part any information which identifies you will be kept confidential and will only be accessible by our researchers and healthcare practitioners involved in your direct care. Any results published will remove your name and any identifiers to ensure this.

Will you contact my GP?

We will only inform your GP of your involvement if you give us permission and only if your test indicates you have hepatitis C.

What are the risks?

Some people may experience slight discomfort when the finger prick is carried out.

You may become distressed if you discover that you have hepatitis C. Anyone whose test indicates they have hepatitis C will be given a follow-up appointment with a specialist nurse where they will receive counselling and further evaluation.

A positive diagnosis may affect the availability, validity or cost of types of insurance, particularly travel insurance.

What are the benefits?

It is generally accepted that it is better to know if you have hepatitis C and/or HIV so you can receive appropriate treatment or monitoring.

PARTICIPANT INFORMATION SHEET 2

The Royal Liverpool Hospital, in collaboration with the University of Liverpool, is trying out new ways to improve care. We would like to ask for your help and feedback with our initiative concerning **hepatitis C and HIV**. We will be offering a screening test for hepatitis C and HIV to all adults attending the A&E Minors department today. Please read the following information carefully.

What is hepatitis C?

Hepatitis C is an infection of the liver caused by the hepatitis C virus.

How do people catch hepatitis C?

The infection is typically transmitted through contact with infected blood. Other infections like HIV can also be transmitted through blood and this is why we are offering both tests together.

How common is hepatitis C?

It is estimated that for every 100 people in Cheshire and Merseyside, four have hepatitis C.

What are the symptoms of hepatitis C?

Hepatitis C remains silent for many years before causing any symptoms and therefore people do not realise that they have the infection unless a test is done. After about 20 years, hepatitis C may cause scarring of the liver (cirrhosis), followed by liver failure requiring a transplant. Cancer of the liver may also develop. It is important to diagnose the infection before these complications occur so that they can be prevented.

Can hepatitis C be treated?

Yes, treatment is available that can cure the infection in over 90% of people. The Royal Liverpool Hospital is one of the major centres in England offering treatment for hepatitis C. Currently, treatment is prioritised according to clinical need.

What is HIV?

HIV is a virus which is carried in the blood. If left untreated, it weakens the immune system and can lead to severe illness and death

The virus is often silent or asymptomatic for periods of time

Can HIV be treated?

Effective treatments now exist for HIV meaning that, with the right treatment and follow-up, life expectancy can be close to normal. We recommend everyone to ‘’know their status’’ so that if diagnosed you can see a specialist and talk about treatment options

How do people catch HIV?

It is usually caught via unprotected sex with an infected person, but can be spread from an infected pregnant woman to a baby or contracted via exposure to blood (eg. Injection from a non-sterile needle or blood transfusion in a country where blood is not screened for HIV)

For more information, you may find these websites useful:

www.nhs.uk/conditions/Hepatitis-C
www.nhs.uk/conditions/HIV

What is this study about?

We are offering adult people attending the A&E department a blood test for hepatitis C and HIV. Our objectives are to encourage people to get tested and be diagnosed, and also to understand what people think about this way of offering a test. Your opinion and feedback are important to us, whether you decide to have the test or not.

How are the hepatitis C and HIV tests done?

Our test uses a few drops of blood collected from a finger prick, instead of blood from a vein. The fingerprick takes 5 minutes. Most people find that it is not painful. The finger prick blood is then tested on a machine in A&E, and the results are ready after 2 hours.

Do I have to wait in A&E for the results?

You may wait if you wish. Alternatively, you can leave your phone number and as soon as the results are ready we will text or call you, depending on your preference.

Do I have to have the test?

We would like to encourage you to have the test. However, you are perfectly free to decline the test. Your decision about the test will not in any way affect the rest of your care today.

What next?

Whether you decide to have a test or not, please could you complete a **short questionnaire** *(see overleaf)*? Your opinion matters to us. It will help us improve the service we offer. The information given in the questionnaire will be completely anonymous and will not be traceable back to you.

What will happen to information stored about me?

If you decide to take part any information which identifies you will be kept confidential and will only be accessible by our researchers or healthcare practitioners involved in your direct care. Any published results will remove your name and any identifiers to ensure you remain anonymous.

Will you contact my GP?

We will only inform your GP of your involvement if you give us permission and only if your test indicates you have hepatitis C/HIV.

What are the risks?

Some people may experience slight discomfort when the finger prick is carried out.

You may become distressed if you discover that you have hepatitis C and/or HIV. Anyone whose test indicates they have hepatitis C will be given a follow-up appointment with a specialist nurse where they will receive counselling and further evaluation.

A positive diagnosis may affect the availability, validity or cost of types of insurance, particularly travel insurance.

What are the benefits?

It is generally accepted that it is better to know if you have hepatitis C and/or HIV so you can receive appropriate treatment or monitoring.

Supplementary Table 1. Study population in validation study, according to known HCV RNA status[a]

	Total	Known HCV RNA status	
		Positive	Negative
Total number (%)	50 (100)	39 (77.4)	11 (22.6)
HCV antibody positive, n (%)	42 (83.0)	39 (77.4)	3 (5.7)[b]
HIV-1 antibody positive, n (%)	16 (30.2)	9 (17.0)	7 (13.2)
HBsAg positive, n (%)	4 (7.5)	1 (1.9)	3 (5.7)

[a]As determined in routine care within the three months prior to study entry; [b]Previous sustained virological response to antiviral therapy. HBsAg= Hepatitis B surface antigen.

Supplementary Figure 1. Bland-Altman analysis of Xpert HCV RNA load results in paired capillary blood and plasma samples. The solid horizontal line represents the mean difference between results. The dotted horizontal lines represent the upper and lower limit of agreement between the specimens (+ and – 1.96 SD). The dashed horizontal line represents the line of equality.

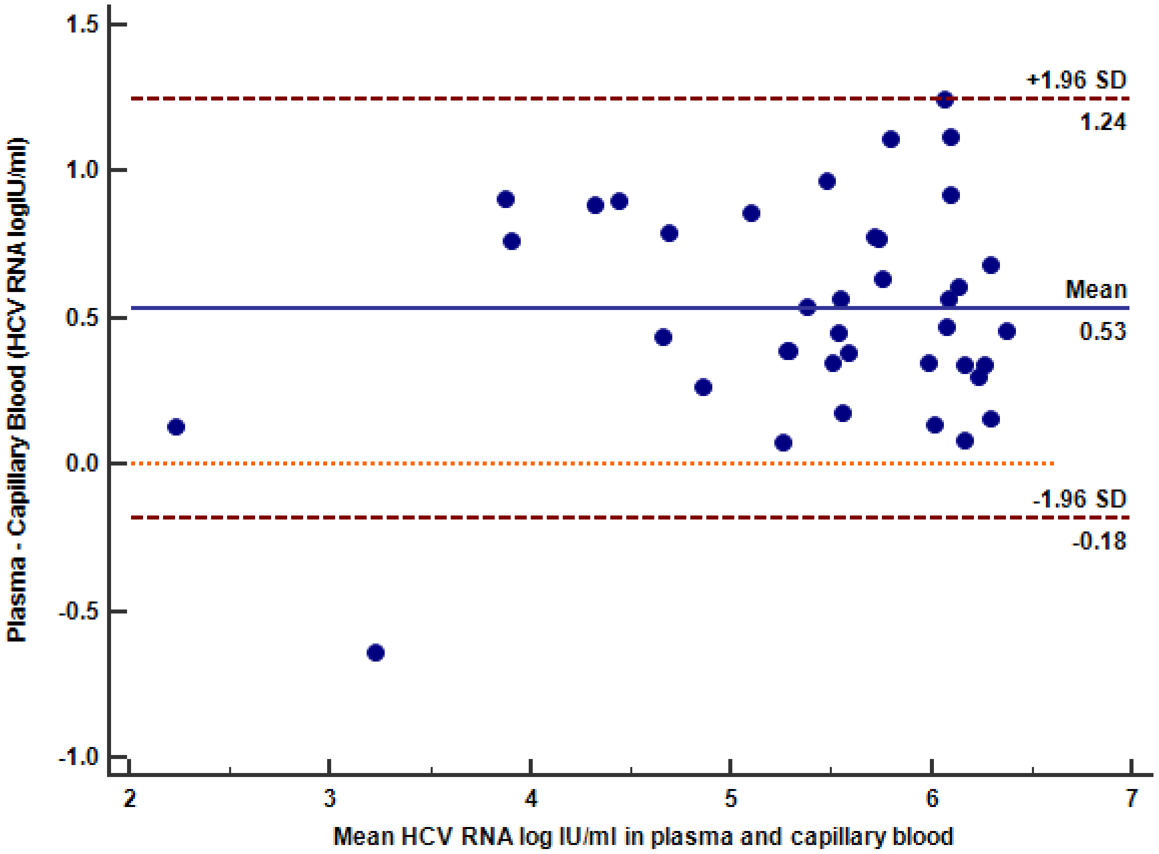

Supplementary Figure 2. Xpert vs. expected HCV RNA load results (log_{10} IU/ml) with a validation panel representing HCV genotypes 1a, 1b, 2b, 3a, 4a, 4d, 5a, and 6a. Serial dilutions (2-5 log_{10} IU/ml) were tested in duplicate according to the small-volume protocol. The lines of equality (diagonal dotted) and Passing-Bablok regression (diagonal solid) bordered by 95% confidence intervals (diagonal dashed) are shown. The theoretical small-volume protocol lower limit of quantitation is represented by the horizontal dashed line. There were 24 samples representing the dilution point of 2 log_{10} IU/ml (actual HCV RNA levels: 140, 149, 100, 120, 108, 110, 290, and 247 IU/ml for genotypes 1a, 1b, 2b, 3a, 4a, 4d, 5a, and 6a, respectively). Of these 24 samples, 12 showed quantifiable HCV RNA levels (median 189 IU/ml; range 145-355 IU/ml)**;** the other 12 samples showed qualitative HCV RNA detection (estimated HCV RNA levels >44 and <110 IU/ml). Median standard deviations (IQR) of duplicate HCV RNA measurements were 0.10 (0.02-0.17), 0.04 (0.03-0.05), 0.07 (0.06-0.07), 0.07 (0.03-0.09), 0.19 (0.12-0.26), 0.05 (0.03-0.06), 0.20 (0.18-0.23) and 0.03 (0.03-0.04) for genotypes 1a, 1b, 2b, 3a 4a, 4d, 5a, and 6a, respectively. R^2 =0.901, y = 0.3339 + 0.9035 x.

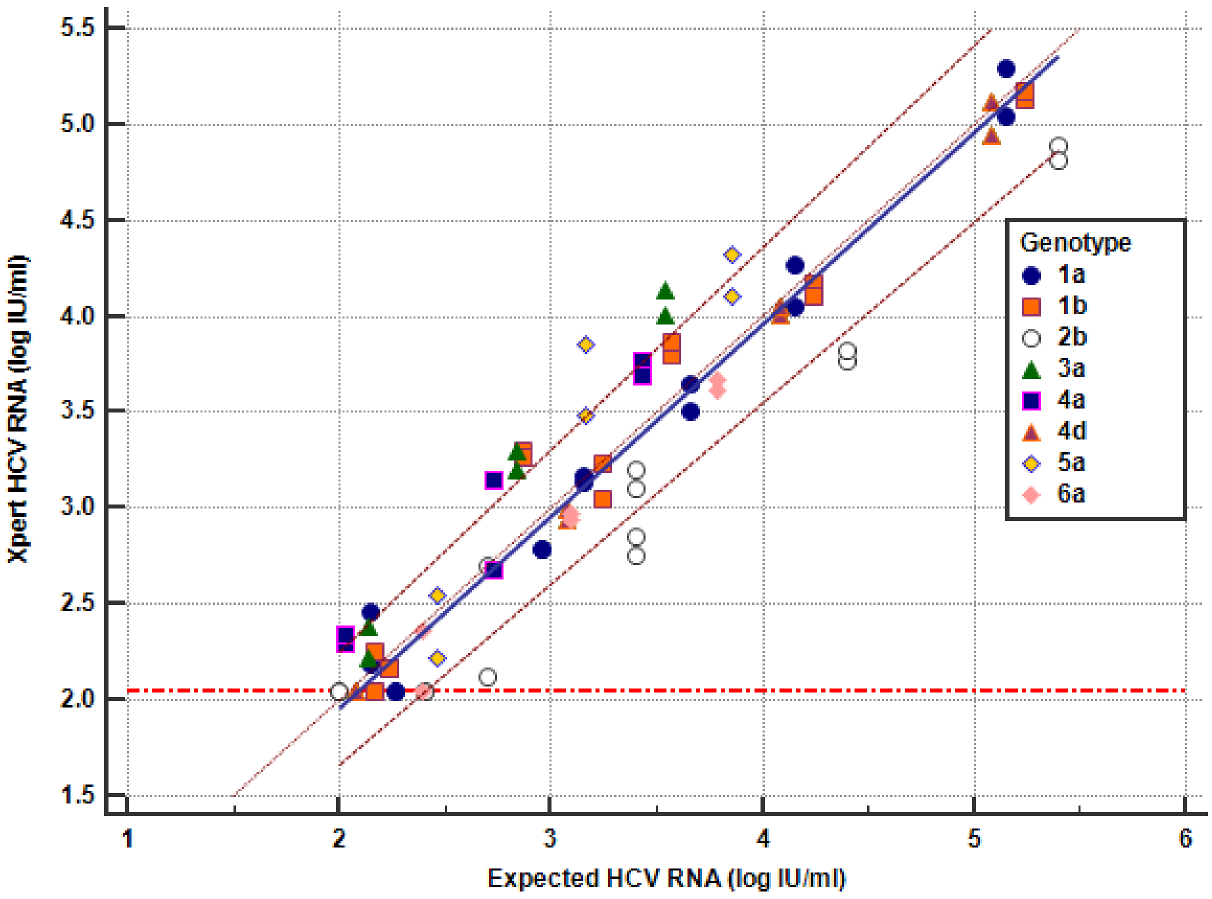

Supplementary Table 2. Deprivation decile rank for POCT participants that indicated their residential postcodes (n=297)

	Decile (n, %)									
Domain of deprivation	1	2	3	4	5	6	7	8	9	10
Income	117 (39.4)	42 (14.1)	20 (6.7)	17 (5.7)	22 (7.4)	25 (8.4)	16 (5.3)	14 (4.7)	11 (3.7)	13 (4.3)
Employment	132 (44.4)	37 (12.4)	16 (5.3)	28 (9.4)	17 (5.7)	21 (7.0)	16 (5.3)	12 (4.0)	7 (2.3)	11 (3.7)
Education Skills & Training	60 (20.2)	65 (21.8)	26 (8.7)	24 (8.0)	18 (6.0)	22 (7.4)	16 (5.3)	29 (9.7)	20 (6.7)	17 (5.7)
Health & Disability	180 (60.6)	41 (13.8)	28 (9.4)	15 (5.0)	9 (3.0)	8 (2.6)	10 (3.3)	3 (1.0)	2 (0.6)	1 (0.3)
Crime	82 (27.6)	43 (14.4)	29 (9.7)	29 (9.7)	24 (8.0)	28 (9.4)	28 (9.4)	9 (3.0)	23 (7.7)	2 (0.6)
Barriers to housing & services	4 (1.3)	12 (4.0)	15 (5.0)	17 (5.7)	16 (5.3)	24 (8.0)	45(15.1)	73 (24.5)	37 (12.4)	54 (18.1)
Living environment	98 (33.0)	40 (13.4)	46(15.4)	41 (13.8)	31(10.4)	17 (5.7)	8 (2.6)	9 (3.0)	5 (1.6)	2 (0.6)
Index of Multiple Deprivation	126 (42.4)	41 (13.8)	22 (7.4)	31 (10.4)	21 (7.1)	16 (5.3)	17 (5.7)	10 (3.3)	8 (2.6)	5 (1.7)

POCT= Point-of-care testing.
